# Animal Chemistry with Reference to the Physiology and Pathology of Man

**Published:** 1846-05

**Authors:** 


### REVIEWS.

---

Art. VI.—*Animal Chemistry with reference to the Physiology and Pathology of Man.* By J. Franz Simon, Fellow of the Society for the advancement of Physiological Chemistry at Berlin, &c., &c.—Translated by Thomas E. Day, M.A. and L.M., Cantab., Licentiate of the Royal College of Physicians. To be complete in 2 parts. Part 1. Philadelphia; Lea & Blanchard. 1845. 12mo. pp. 292.

We closed our former article on this work with a table of the constituents of the blood as affected by pneumonia biliosa, in a case of which it was shown that the fibrin had risen to the extraordinary height of 12·72 in a thousand parts.

The author next gives the results obtained by Andral and Gavarret in their analysis of pleuritic blood. The increase in the fibrin was found by these chemists to be from 3, the healthy standard, to 4, 5·9, and 6, according to the stage of the disease, being greatest when the pleuritis was not yet advanced, but where effusion had taken place, and less in the more advanced periods of the affection. In five cases of pleuritis Becquerel and Rodier found the mean proportion of fibrin to be 6·1.

In peritonitis the increase in the fibrin was not observed by the author to be so great as in pleuritis, but still in that form of the complaint denominated puerperal fever he found that it amounted to twice as much as in healthy blood. Andral and Gavarret found it even as high as 6·1 and 7·2.

"In a woman, aged 24 years, attacked with metroperitonitis, Scherer observed a tolerably large buffy coat, apparently more gelatinous than tough. The clot was rather large, but not very firm. The serum was neutral.

The blood contained in 1000 parts:

| | |
|---|---|
| Water, | 814·53 |
| Solid constituents, | 185·47 |
| Fibrin, | 5·32 |
| Albumen, | 96·35 |
| Blood-corpuscles, | 70·16 |
| Fat and extractive matters, | 6·02 |
| Salts, | 7·13 |

Two days after the blood contained:

| | |
|---|---|
| Water, | 832·58 |
| Solid constituents, | 167·42 |
| Fibrin, | 4·02 |
| Albumen, | 100·25 |
| Blood-corpuscles, | 52·30 |
| Salts and extractive matters, | 11·42 |

The buffy coat had a more gelatinous appearance, and the serum was redder than on the former occasion. Death occurred two days after the second venesection."

The same increase of fibrin was noted in inflammation of the uropoietic viscera. In a case of cystitis the blood examined by Andral and Gavarret gave 5·4 of fibrin. Rheumatism develops this principle very sensibly, and the increase seems to bear some proportion to the intensity of the pain. Thus, says our author:

"The blood will be found to contain as large a proportion of fibrin at the commencement of a rheumatic attack which begins very severely, as at a much later period in a case commencing mildly, but in which acute pain gradually supervenes. This will be seen from the following analyses:

| | Venesection. | Day of disease. | Water. | Fibrin. | Blood-corpuscles. | Residue of serum. |
|---|---|---|---|---|---|---|
| 1st Case, | 1 | 4 | 797·1 | 8·9 | 109·3 | 84·7 |
| | 2 | 5 | 796·9 | 9·8 | 107·5 | 81·8 |
| | 3 | 6 | 812·5 | 8·5 | 95·4 | 83·6 |
| | 4 | 10 | 820·6 | 6·4 | 93·5 | 79·5 |
| | 5 | 25 | 789·7 | 2·8 | 117·9 | 89·6 |

| | Venesection. | Day of disease. | Water. | Fibrin. | Blood-corpuscles. | Residue of serum. |
|---|---|---|---|---|---|---|
| 2d Case, | 1 | 8 | 778·8 | 6·1 | 123·1 | 92·0 |
| | 2 | 9 | 780·9 | 7·2 | 120·7 | 91·2 |
| | 3 | 10 | 788·0 | 7·8 | 112·8 | 91·4 |
| | 4 | 13 | 799·0 | 10·2 | 101·0 | 89·8 |
| | 5 | 17 | 813·9 | 9·0 | 89·2 | 87·9 |
| | 6 | 28 | 826·2 | 7·0 | 83·8 | 83·0 |

"In the first case, the maximum of fibrin is found in the blood taken at the second venesection, and as early as the fifth day of the disease. In the second case, on the contrary, it did not occur until the fourth venesection, upon the thirteenth day of the disease, when nearly all the joints were reported to be in a swollen and painful state. These symptoms began to diminish after the next two bleedings; the fever, however, still continued."

In erysipelas the fibrin has been found in as large a proportion as 7·3, while in milder cases of the malady, attended with but little fever, it has amounted to only 3·6. The quantity seems to be determined rather by the severity than the extent of the affection, for in a case of erysipelas of the hand the blood yielded 7·71 of fibrin. In one of the face, it was only 5·45.

The blood of phthisical patients is inflammatory. The clot is small, consistent, and covered with a buffy coat; the serum is clear, and of a bright yellow color, during one period of the disease; but the blood differs considerably in the progressive stages of consumption. As the tubercles soften the quantity of fibrin increases to 4·5, in some instances; and when vomicæ form in the lungs may rise to 5·5, or even 5·9, but never attains the height observed in pneumonia. In the very last stage of the disease, as the blood becomes poor, the fibrin diminishes, and even falls below the healthy standard. As the changes in the blood attendant upon phthisis are particularly interesting we copy from the author the following tables and observations:

"I have made three analyses of the blood of phthisical persons, the results of which are not devoid of iuterest.

| | Analysis 26. | Analysis 27. | Analysis 28. |
|---|---|---|---|
| Water, - - - | 807·500 | 825·200 | 750·000 |
| Solid residue, - - | 192·500 | 174·800 | 250·000 |
| Fibrin, - - - | 4·600 | 6·500 | a trace. |
| Fat, - - - - | 2·350 | 4·200 | 3·750 |
| Albumen, - - - | 98·360 | 90·350 | 131·000 |
| Globulin, - - - | 71·230 | 61·110 | 94·500 |
| Hæmatin, - - - | 3·110 | 2·690 | 2·750 |
| Extractive matters and salts, | 9·350 | 8·000 | 15·250 |

"The blood in analysis 26 was taken from a man aged 36 years, in the second stage of tubercular phthisis, who afterwards sunk under the disease. The blood in analysis 27 was taken from a man aged 41, in the third stage of the disease, who suffered extremely from nocturnal colliquative sweats, and from feverish symptoms. In these two instances the blood exhibits the characters of hyperinosis, for the quantity of fibrin is in one instance twice, and in the other thrice the normal amount, and the amount of hæmato-globulin is below the healthy standard: moreover, the quantity of solid constituents is less than in healthy blood. Andral and Gavarret's observations respecting the changes that the blood undergoes as the disease advances are here borne out.

"The 28th analysis gives results quite at variance with the two former. The blood in this instance was taken from a man about 30 years of age, who was treated in our hospital for tubercular phthisis. He had taken cod-liver oil for some-time with much benefit; subsequently, however, frequent attacks of hemoptysis came on, for which venesection was always immediately prescribed. The clot in these cases was seldom very firm. I analysed the blood taken at his last venesection. It was received into a shallow vessel, and amounted to between six and seven ounces. It did not coagulate, and it presented the appearance of a homogeneous dark red fluid, in which some white gelatinous flocks of coagulated fibrin were swimming.

"The blood contained, much to my surprise, a larger amount of solid constituents than I have ever observed in any other analysis. The fat, when isolated, smelt strongly of the volatile fatty acid of the cod-liver oil, the odor of which was also strongly developed during the evaporation of the blood to dryness. A considerable quantity of hæmaphæin was present, and deeply colored the extractive matters and salts. It is very probable that the peculiar changes in the blood in this instance are due principally to the cod-liver oil and to the repeated bleedings."

The opposite state of the blood to the one we have been considering, is styled by Dr. Simon *hypinosis;* and this is exhibited in typhus, intermittent and continued fevers, variola, and varioloid, rubeola, and, according to our author, in scarlatina. This is more than we were prepared to expect. Assuredly in scarlatina we should have looked for an increase in the amount of fibrin; and in some of the analyses of Andral and Gavarret this was the case. In two they found it nearly normal in quantity, in one 4·0, and in one even as high as 5·8. But scarlet fever is a disease the character of which varies exceedingly in different constitutions, and during different epidemics. It is therefore difficult to assign to the blood in that complaint any specific condition. In one case it might exhibit even a deficiency of fibrin, while in most others the blood would show evident signs of inflammation. In typhus the fibrin is for the most part normal in quantity, very seldom, and then only slightly, above the mean, and often decidedly less than it is found in healthy blood. It fell as low as 1·6 in one case cited by Dr. Simon. In measles it varied from 2·4 to 3·4, being little altered from its natural state. In intermittent fever its maximum, in the cases investigated, was 3·8 and its minimum 3·0. The author thus sums up:

"The composition of the blood in hypinosis is essentially the reverse of that in hyperinosis. The amount of corpuscles is increased, that of fibrin is diminished, and the solid constituents generally are increased rather than diminished; while in the phlogoses they are most commonly below the normal standard. We have seen in the previous analyses that in proportion as the febrile symptoms assumed the form of erethismus, the characters of hypinosis became less marked; and, on the other hand, that when they took on a torpid type these characters were more strikingly developed."

The third form of diseased blood described by Dr. Simon he styles "*spanæmia*," the meaning of which is *poor blood*, the vital fluid being in that case especially deficient in fibrin and blood-corpuscles. Some of the diseases in which this condition is presented are cancer, scrofula, chlorosis, purpura hemorrhagica, and petechial typhus, all of which are characterized by anemia. It is the experience of the author, in which he supports Andral and Gavarret, that the solid constituents of the blood, and especially the globulin, are increased by ferruginous preparations. Confirmatory of this fact Dr. Simon gives the following tables, the first showing the constitution of the blood of a chlorotic girl when first subjected to treatment; the second exhibiting it at the end of seven weeks, during which time she had taken two ounces of the tincture of iron, and sixty-four grains of metallic iron.

| | Analysis 32. | Healthy blood. |
|---|---|---|
| Water, - - - - | 871·500 | 795·278 |
| Solid constituents, - - | 128·500 | 204 022 |
| Fibrin, - - - - | 2 080 | 2 104 |
| Fat, - - - - - | 2·520 | 2·346 |
| Albumen, - - - - | 79 820 | 76 660 |
| Globulin, - - - - | 30·860 | 103 022 |
| Hæmatin, - - - - | 1·430 | 6 209 |
| Extractive matters and salts, | 11·000 | 12 012 |

The hæmato-globulin contained 4·4$\frac{0}{0}$ of coloring matter.

| | Analysis 33. |
|---|---|
| Water, - - - - - | 806 500 |
| Solid residue, - - - - | 193 500 |
| Fibrin, - - - - - | 1·200 |
| Fat, - - - - - - | 2·299 |
| Albumen, - - - - - | 81·230 |
| Globulin, - . . - - | 90 810 |
| Hæmatin, - - - - - | 4·598 |
| Extractive matters and salts, - | 9 580 |

The hæmato-globulin contained 4 8$\frac{0}{0}$ of coloring matter.

Purpura hemorrhagica, though placed in this connexion, does not appear to exhibit the characters of spanæmia, but shows, as will be seen by the following analysis, a great deficiency of fibrin.

| | |
|---|---|
| Water, - - - - - - | 795·244 |
| Solid constituents, - - - | 204·756 |
| Fibrin, - - - - - - | 0 905 |
| Blood-corpuscles, - - - | 121·701 |
| Residue of serum, - - - | 83·405 |

The fourth form of diseased blood our author calls *hetero-chymensis*, upon which he makes the following explanatory remarks:

"I arrange under this form all those states of the blood in which a substance is present that does not exist in the normal fluid: when, for instance, the blood contains urea (in appreciable quantity,) sugar, coloring matter of the bile, fat, pus. The circumstances that lead to the establishment of this diseased condition of the blood are far less natural than those which are connected with the production of the three former classes. The arrangement is artificial, and merely adopted for convenience, since this class of disease has simply this property in common, that the composition of the blood is here *qualitatively* changed, whilst in the three former it is only altered *quantitatively.* The putrid form of typhus, the yellow fever, and the plague, certainly might have been placed in the class, since coloring matter of the bile, and a salt of ammonia, are often found in the serum. I have, however, thought it best to place these diseases in the third class, because, in the first place, the presence of the abnormal constituents is not constant; and because, secondly, in consequence of the deficiency in the solid constituents of the blood in these disorders, they naturally occur under the class spanæmia."

In the *morbus Brightii* the result of numerous analyses indicates a decreased quantity of solid constituents, a small amount of blood-corpuscles, and the presence of *urea* in the blood. *Sugar* has been detected in the blood of diabetic patients; *bile* appears in some of the affections of the liver; and according to Gulliver, *pus* is "found in the blood in all diseases in which there is suppuration, or even inflammatory swelling, accompanied with hectic fever." But, worse than all, this fluid, it seems, is sometimes infested with *animals.* Our author informs us that

"Dr. Chiaje, of Naples, has recently stated that he found the polystoma sanguiculum in the expectorated blood of two phthisical patients who were attacked with hæmoptysis.—Some of these small flat worms, which are similar to leeches, were floating about in the serum, others attached themselves to the sides of the vessels."

The London Lancet, for 1840, gives similar cases. Dr. Goodfellow found an immense number of animalculæ in the blood of a fever-patient. In the blood of a boy laboring under influenza Mr. Bushman saw "*worms half an inch long.*"

"Can such things be,
And o'ercome us like a summer's cloud,
Without our special wonder!"

From a number of analyses of the blood of *pregnant women* it would appear that the composition of that fluid is materially affected by pregnancy. In density it is diminished; the water, the fibrin, and the phosphorized fat are increased, while the albumen and corpuscles are diminished. The menstrual blood was subjected to analysis by Becquerel and Rodier, and the results are stated by Dr. Simon in the following paragraph:

"The most striking peculiarities of this blood are, the total absence of fibrin, and the increase of the solid constituents caused by the excess of the blood-corpuscles. The hæmato-globulin was found to be very rich in hæmatin, combined, undoubtedly, with a considerable amount of hæmaphæin; the coloring matter amounted to 8·3% of the hæmato-globulin."

The blood of the lower animals has been investigated with great care by several chemists, and the chief results are quoted at the close of this volume, where an account is also given of lymph and chyle. We shall not follow him into these details having now presented so much of the work to the readers of the Journal as will enable them to judge of its character. As a repository of facts in animal chemistry, no volume yet published in our language can compare with it.

